# Beneficial Roles of Melatonin on Redox Regulation of Photosynthetic Electron Transport and Synthesis of D1 Protein in Tomato Seedlings under Salt Stress

**DOI:** 10.3389/fpls.2016.01823

**Published:** 2016-11-30

**Authors:** Xiaoting Zhou, Hailiang Zhao, Kai Cao, Lipan Hu, Tianhao Du, František Baluška, Zhirong Zou

**Affiliations:** ^1^College of Horticulture, Northwest A&F UniversityYangling, China; ^2^Key Laboratory of Protected Horticultural Engineering in Northwest, Ministry of AgricultureYangling, China; ^3^Institute of Cellular and Molecular Botany, University of BonnBonn, Germany

**Keywords:** *Solanum lycopersicum*, salt stress, redox regulation, melatonin, photosynthetic characteristics

## Abstract

Melatonin is important in the protection of plants suffering various forms of abiotic stress. The molecular mechanisms underlying the melatonin-mediated protection of their photosynthetic machinery are not completely resolved. This study investigates the effects of exogenous melatonin applications on salt-induced damage to the light reaction components of the photosynthetic machinery of tomato seedlings. The results showed that melatonin pretreatments can help maintain growth and net photosynthetic rate (PN) under salt stress conditions. Pretreatment with melatonin increased the effective quantum yield of photosystem II (ΦPSII), the photochemical quenching coefficient (qP) and the proportion of PSII centers that are “open” (qL) under saline conditions. In this way, damage to the photosynthetic electron transport chain (PET) in photosystem II (PSII) was mitigated. In addition, melatonin pretreatment facilitated the repair of PSII by maintaining the availability of D1 protein that was otherwise reduced by salinity. The ROS levels and the gene expressions of the chloroplast TRXs and PRXs were also investigated. Salt stress resulted in increased levels of reactive oxygen species (ROS), which were mitigated by melatonin. In tomato leaves under salt stress, the expressions of PRXs and TRXf declined but the expressions of TRXm1/4 and TRXm2 increased. Melatonin pretreatment promoted the expression of TRXf and the abundances of TRXf and TRXm gene products but had no effects on the expressions of PRXs. In summary, melatonin improves the photosynthetic activities of tomato seedlings under salt stress. The mechanism could be that: (1) Melatonin controls ROS levels and prevents damaging elevations of ROS caused by salt stress. (2) Melatonin facilitates the recovery of PET and D1 protein synthesis, thus enhancing the tolerance of photosynthetic activities to salinity. (3) Melatonin induces the expression of TRXf and regulates the abundance of TRXf and TRXm gene products, which may facilitate repair of the light reaction parts of the photosynthetic machinery.

## Introduction

In many parts of the world, salt-affected soils cause considerable economic loss through unacceptable levels of yield reduction. High salt levels cause both secondary osmotic stress and ion toxicity which together suppress many key biochemical processes (Zhu, 2002). When plants are exposed to salt stress, their photosynthetic activity is significantly depressed. Down-regulation of CO_2_ fixation by salt stress can lead to problems with excessive light energy and the accumulation of reactive oxygen species (ROS) (Takahashi and Murata, 2008; Tikkanen et al., 2014). Within the photosynthetic machinery, PSII is particularly sensitive to such abiotic stresses. For example, ROS damages the photosynthetic apparatus (Hu et al., 2014) and blocks the synthesis of PSII proteins in the chloroplasts (Nishiyama et al., 2011). In particular, PSII is rapidly inactivated under strong-light conditions—referred to as PSII photoinhibition. However, a repair system continuously restores PSII activity, so that photoinhibition of PSII becomes apparent only when the rate of light-induced inactivation exceeds the rate of repair. Salt stress increases the severity of photoinhibition (Ohnishi and Murata, 2006) because turnover of D1 protein (an integral part of the reaction center of PSII) is inhibited under salt stress. Studies have shown that salt stress impedes D1 protein synthesis by direct inactivation of the translation machinery at translation level (Allakhverdiev et al., 2002; Nishiyama and Murata, 2014). Therefore, to improve PSII tolerance to abiotic stress, it will necessary somehow to increase the rate of PSII repair.

Photosynthetic organisms have developed many strategies to adapt to a fluctuant environment. The light-induced photosynthetic process is usually connected with electron transport efficiency (Nishiyama and Murata, 2014). Environmental changes have direct and indirect effects on the redox state of the photosynthetic electron transport (PET) components and on the redox-active compounds (TRXs or glutathione) through their effects on electron transport efficiency (Dietz and Pfannschmidt, 2011). The redox state of the aqueous phase is controlled by soluble redox metabolites which include NAD(P)H and glutathione, metabolite pairs such as malate and oxaloacetate, and other thiol/disulfide proteins such as TRXs and PRXs (Foyer and Noctor, 2009). The efficiency of photosynthesis is enhanced by regulating environmentally induced changes in the redox state of photosynthetic components or by the generation of ROS (Takahashi and Murata, 2008). For instance, salt stress reduces the activity of the Calvin-Benson cycle, which in turn results in reduced production of the last electron receptor, NADP^+^ in photosystem I (PSI) and the PET chain. Generally, electrons are transferred from PSII to PSI, electron end acceptor NADP^+^ accepting electrons via ferredoxin (Fd). However, excess electrons can also be transferred to oxygen in PSI or via the Mehler reaction which generates ROS (Foyer and Noctor, 2009). In contrast, a balance between ROS production and ROS scavenging in the chloroplast is essential for photosynthetic function because of oxidative damage by ROS. There are many strategies for controlling ROS levels in the chloroplast. These include the ascorbic acid—glutathione (AsA-GSH) cycle and redox regulatory components such as TRXs and PRXs (Foyer and Noctor, 2009). The AsA-GSH cycle, together with superoxide dismutase (SOD) and ascorbate peroxidase (APX) are efficient at metabolizing ROS. However, it has been suggested the later scavengers of ROS, TRXs, and PRXs, participate in redox regulation in many physiological process. The PRXs reduce H_2_O_2_ through a peroxidative reduction, followed by a regeneration that can involve a variety of electron donors such as TRXs, glutaredoxin, cyclophilins, GSH, and AsA. In addition, several of these key regulatory redox-reactive molecules, such as TRXs, serve as signal molecules that activate or inactivate the expression of chloroplast and nuclear genes, essential for regulating most plastid processes, such as: carbohydrate and lipid metabolism, and also gene transcription and protein synthesis (Dietz and Pfannschmidt, 2011). The ROS-induced suppression of the *de novo* synthesis of proteins, such as D1 protein, thus inhibit the moieties required for PSII repair (Nishiyama et al., 2011). The translation of D1 protein is regulated by the redox state of translation elongation factor G (EF-G) in *Synechocystis* sp. PCC 6803, which is oxidized by ROS while reduced by TRXs, an electron donor in the photosynthetic transport chain (Kojima et al., 2009). It has recently been suggested the redox regulation of translation elongation could be a control strategy in higher plants (Moore et al., 2016).

Melatonin is an indoleamine synthesized in plants that has been shown to play important roles in the circadian growth rhythms and stress responses (Arnao and Hernandez-Ruiz, 2014). Previous studies show that melatonin functions as a potent antioxidant and scavenger of free radicals (Zhang and Zhang, 2014; Manchester et al., 2015). It also upregulates anti-stress genes involved in the regulation of photosynthesis, the cell cycle, DNA replication, carbohydrate metabolism, and lipid biosynthesis under abiotic stress conditions (Arnao and Hernandez-Ruiz, 2014). In particular, melatonin plays a recovery role in photosynthesis under environmental stress. Melatonin improves the functions of stomata, through the maintenance of the water balance and down-regulated the ABA pathway in *Malus hupehensis* through under drought stress (Li et al., 2015). Melatonin contributes to the efficiency of electron transport under conditions of water deficiency (Wang et al., 2013; Zhang et al., 2013; Meng et al., 2014). However, to avoid damage from excess electrons in the chloroplast, (e.g., ROS), melatonin plays a protective role by directly scavenging ROS and also by up-regulating the antioxidant enzymes and antioxidants (Arnao and Hernández-Ruiz, 2015; Li et al., 2016). Meanwhile, less research attention has been paid to cross-talk between melatonin and TRXs and PRXs, which also play important functional roles in redox regulation. Redox regulation of protein synthesis also merits further research with respect to the effects of melatonin.

In this study, we analyze the effects on PET of exogenous melatonin supplied to the soil (roots) every 6 days under salt stress conditions. We also evaluate redox regulation by the TRXs, to explore the effects of melatonin on the synthesis of critical proteins, using PSII repair under salt stress as an example. Our aim was determined whether increases in photosynthesis under salt stress following melatonin supplement are related to melatonin-induced protection of the photosynthetic machinery. It was also considered useful to clarify relationships between melatonin and the redox-regulated components such as TRXs in the repair of PSII.

## Materials and methods

### Plant materials and treatments

The experiments were conducted under controlled environment conditions with relative humidity (RH) in the range 60–80%, day/night temperatures of 25/15°C, a photoperiod of 16/8 h, and a daytime photosynthetic photon flux density of 350 μmol m^−2^ s^−1^. Tomato seedlings (*Solanum lycopersicum* cv. Jin Peng Yi Hao) bearing three true leaves (35 days after germination) were transplanted into black plastic pots (15 × 15 cm) filled with planting medium (Floragard, Germany; PH, 5.6; N, 140 mg/L; P_2_O_5_, 80 mg/L; K_2_O, 190 mg/L).

To investigate the influence of melatonin on chlorophyll fluorescence in tomato plants under salt stress, the roots of tomato seedlings at the three-leaf stage were pretreated with exogenous melatonin irrigated into the soil at concentrations of: 0, 1, 50, 100, 150, and 200 μM melatonin. The melatonin-pretreated seedlings were then treated with 150 mM NaCl contained in Hoagland's nutrient solution, while the same melatonin treatments were repeated every 6 days. The zero NaCl, zero melatonin controls were irrigated with Hoagland's nutrient solution without additions of melatonin or NaCl. Based on these experiments, irrigation with 150 μM melatonin was selected as optimal for studying the effects of exogenous melatonin on the NaCl stress response.

In the main experiment, plants and treatment applications were the same as above. The control and three treatments were: CK (control)—Hoagland's solution alone; M (melatonin)—Hoagland's solution containing 150 μM melatonin; S (salt)—Hoagland's solution containing 150 mM NaCl; and M+S (melatonin + salt)—Hoagland's solution containing 150 μM melatonin and 150 mM NaCl.

On three occasions after treatment, i.e., at day 0, day 1, and day 12 (plants still vegetative), the second fully-expanded leaf (counting from the top) was sampled from each of three replicate plants from each treatment. For whole plant dry weight (shoot + root), the planting medium was carefully washed away from the roots, the plants were immediately deactivated in an oven at 105°C for 15 min and then dried to constant weight at 60–80°C for 24–48 h.

### Analysis of gas exchange parameters, and chlorophyll fluorescence

The gas exchange parameters were measured using a portable photosynthesis system (LI-6400, LI-COR Inc., USA). These parameters included: net photosynthetic rate (PN), stomatal conductance (Gs), intercellular CO_2_ concentration (Ci), and transpiration (Tr). In the assimilation chamber, leaf temperature was maintained at 25°C, RH in the range 60–80%, external CO_2_ concentration at 600 ± 10 μmol mol^−1^ and light intensity was constant at 600 μmol photons m^−2^s^−1^.

Chlorophyll fluorescence was measured after dark adaptation for 30 min, according to the method previously described (van Kooten and Snel, 1990). For this a portable fluorometer (PAM 2500; Walz, Germany) was used, selecting the second fully-expanded leaf from the top of the plant. The PSII maximum quantum yield (Fv/Fm) was determined after dark adaptation for 30 min. The initial chlorophyll fluorescence yield (Fo) was determined under low-modulated measuring light (<0.1 μmol m^−2^s^−1^), followed by a 0.8 s pulse of saturating white light (8000 μmol m^−2^s^−1^) to obtain maximum fluorescence yield (Fm). To determine the minimal fluorescence level in a leaf during the illumination (Fo′) and to allow maximal oxidation of the PSII centers in the presence of far red light, a black cloth was rapidly placed so as to block out light by covering the leaf and the leaf-clip holder. Immediately after light blockage, leaf fluorescence fell to the Fo′ level but rose again after a few seconds. The steady-state fluorescence levels (Fs) and the maximum fluorescence levels (Fm′) during light exposure were also measured. Fluorescence levels were used to calculate the effective quantum yield of PSII photochemistry [ΦPSII = (Fm′ − Fs)/Fm], the effective quantum use efficiency of PSII [Fv′/Fm′ = (Fm′ − Fo′)/ Fm′], the photochemical quenching coefficient [qP = (Fm′ − Fs)/(Fm′ − Fo′)], the fraction of PSII centers that are “open” [qL = qP^*^ (Fo′/Fs)], and the non-photochemical quenching [NPQ = (Fm − Fm′)/Fm′].

### Histochemical localization of ${\text{O}}_{2}^{-}$ by NBT staining and H_2_O_2_ by DAB staining

Nitroblue tetrazolium (NBT) staining was used for histochemical localization of ${\text{O}}_{2}^{-}$ after Kim et al. (2011), with some modification. Leaves were incubated in HEPES buffer (pH 7.5) containing 6 mM NBT. Leaves were vacuum infiltrated gently for 1 min, then shaken in the dark for 2 h (80 rpm/min). After this, the NBT staining solution was replaced by 95% (v/v) ethanol. Chlorophyll was removed by boiling in 95% (v/v) ethanol for 10 min. Insoluble, dark blue formazan compounds were formed by the superoxide accumulation.

The H_2_O_2_ was measured using 3,3′-diaminobenzidine (DAB) staining. Leaves were incubated for 5 min in a solution of 1 mg/mL DAB, under a gentle vacuum for infiltration. They were then shaken for 5 h in the dark (80 rpm/min). Next, the DAB staining solution was replaced by a bleaching solution (ethanol:acetic acid:glycerol; 3:1:1) and the chlorophyll removed by boiling for 15 min. The DAB forms a brown precipitate in reaction with hydrogen peroxide.

### Total RNA extraction and gene expression analysis

Total RNA was isolated from leaves using the E.Z.N.A. Plant RNA Kit (Omega Bio-tek, Doraville, GA, USA) according to the manufacturer's instructions. Reverse transcription was carried out using a PrimeScript™ RT reagent kit with gDNA Eraser (Takara, Shiga, Japan) in a 20-μL reaction mixture containing 1 μg of total RNA from each sample. These cDNAs were used as templates for qRT-PCR. The real-time PCR assay was carried out on a CFX96™ real-time PCR cycler (Bio-Rad, Hercules, CA, USA). An initial denaturation at 95°C for 30 s was followed by 40 cycles at 95°C for 5 s, 60°C for 34 s and a melt curve 65–95°C using the SYBR Premix Ex TaqTM II (Tli RNaseH Plus) Kit (Takara, USA). Actin gene was used as internal standard.

Primers for actin was followed the designing as described by Wu et al. (2014). Primers for genes of TRXf and TRXm1/4 and TRXm2 were designed as described by Cheng et al. (2014). Primers for FTR, NTRC, PRXQ, PRX2E-1, PRX2E-2, 2CPA, and 2CPB were designed using Primer 3 version 4.0.0 (website software) with the setting of primer length of 19–24 bp, a melting temperature 55–62°C, a GC content 30–70% and product size 150–250 bp. (see Supplementary Table 1, Supporting Information).

### Protein extraction and western blotting

Protein extraction for TRXf and TRXm followed the method of Fernández-Trijueque et al. (2012) for total protein extraction, with electrophoresis in polyacrylamide gel (Laemmli, 1970). Western-blotting experiments were carried by using specific antibodies against pea PsTRXf and PsTRXm due to sequence similarity of TRXf and TRXm between tomato and pea (Supplementary Figure 2). Polyclonal antibodies against pea-leaf chloroplastic TRX-f and pea-leaf chloroplastic TRXm were used, both being diluted 1:1000 (v/v). Samples (50 μg) of protein were loaded into each lane from tissue-protein extracts.

Western blot analysis of D1 protein using polyclonal antibody (Agrisera, Sweden) and secondary antibody (anti-rabbit IgG horse-radish peroxidase conjugated, Agrisera, Sweden). Soluble protein was extracted, quantified and analyzed by western blot (Hu et al., 2016). Experimental conditions were identical in all three experiments.

### Statistical analyses

Data were analyzed with SAS statistical software (version 8.0, SAS Institute, Cary, NC, USA) using ANOVA and Duncan's multiple range test (*P* < 0.05). Each treatment value was at least in triplicate.

## Results

### Effects of NaCl on chlorophyll fluorescence parameters with and without melatonin

Compared to the controls, NaCl significantly decreased ΦPSII, Fv′/Fm,′ qP and qL while increasing NPQ. This indicates enhanced thermal energy dissipation in PSII (Table 1). However, when the NaCl-stressed plants received different concentrations (1, 50, 100, 150, and 200 μM) of melatonin, those at the higher concentrations, especially 150 μM, showed significant, ameliorative increases for ΦPSII (32%), qP (32%) and qL (40%), compared with NaCl alone. Compared with NaCl alone, these values were also increased slightly with 50 and with 100 μM melatonin; ΦPSII by 14% and 18%, qP by 25% and 17% and qL by 42% and 19%, respectively. Compared with salt alone, there were no significant changes with melatonin treatment in Fv′/Fm′ or NPQ. Treatment with lower concentrations of melatonin (1 μM) showed no significant change compared to with NaCl alone, nor with the higher concentration of melatonin (200 μM) compared to with NaCl alone. The results indicate a concentration effect with increasing levels of melatonin, which rises to a peak at 150 μM melatonin, but falls away rapidly for melatonin concentrations above 150 μM.

**Table 1:** **Effects of NaCl with and without melatonin on chlorophyll fluorescence parameters in tomato seedlings**.

**Treatment**	**Fv′/Fm′**	**ΦPSII**	**qP**	**q L**	**NPQ**
CK	0.6737±0.0173a	0.2736±0.0235a	0.4052±0.0274a	0.1818±0.0134abc	0.9247±0.1466d
S	0.5347±0.0045b	0.1616±0.0105cd	0.3019±0.0180bc	0.1677±0.0109bc	2.0111±0.0812abc
M1+S	0.5103±0.0156bc	0.1367±0.0071d	0.2683±0.0165c	0.1527±0.0137bc	1.9760±0.0994bc
M50+S	0.5438±0.0123c	0.1839±0.0143bc	0.3771±0.0334ab	0.2377±0.0288a	2.3215±0.0749a
M100+S	0.5362±0.0046b	0.1913±0.0168bc	0.3519±0.0313ab	0.1995±0.0227ab	1.7709±0.0296c
M150+S	0.5361±0.0200b	0.2131±0.0109b	0.3973±0.0096a	0.2339±0.0089a	1.9637±0.1302bc
M200+S	0.5003±0.0137bc	0.1237±0.0115d	0.2472±0.0210c	0.1412±0.0136c	2.1551±0.0972ab

*The data were obtained for the first expanded leaves following 12 days of salt stress. Values represent the means ± SE (n = 3). Letters indicate significant differences at P < 0.05 according to Duncan's multiple range tests. Treatments: CK (control), zero NaCl + zero melatonin; S, zero melatonin + 150 mM NaCl; M1+S, 1 μM melatonin + 150 mM NaCl; M50+S, 50 μM melatonin + 150 mM NaCl; M100+S, 100 μM melatonin + 150 mM NaCl; M150+S, 150 μM melatonin + 150 mM NaCl; M200+S, 200 μM melatonin + 150 mM NaCl*.

### Partitioning of absorbed light energy

**Figure 1:**
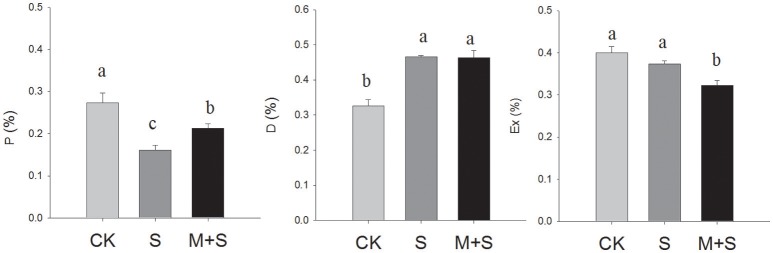
**Partitioning of absorbed light energy in tomato seedlings**. P, reduced PSII photochemistry; D, antenna heat dissipation; Ex, excess energy in the reaction center during the photochemical reaction. The results were calculated from data for 12-day, salt-stressed leaves. CK, control; S, 150 mM NaCl; M+S, 150 μM melatonin with 150 mM NaCl. Values represent the means ± SE (*n* = 3).

For treatment with 150 μM of exogenous melatonin under salt stress, significant differences in the distribution of light energy were detected among treatments (Figure 1). Light energy absorbed by the plant can be dissipated in three ways: (1) in the PSII photochemistry (P), (2) in antenna heat dissipation (D) and (3) in excess energy in the reaction center (Ex). The NaCl stress decreased the P component and increased the D component, while not changing the Ex component. However, under NaCl stress in the presence of melatonin, P increased and Ex decreased (compared with the NaCl- stressed plants) with no significant change in D. The results indicate that under salt stress, melatonin pretreatment alleviated P and lowered Ex.

### Effects of NaCl with or without melatonin on gas exchange parameters and plant growth

**Figure 2:**
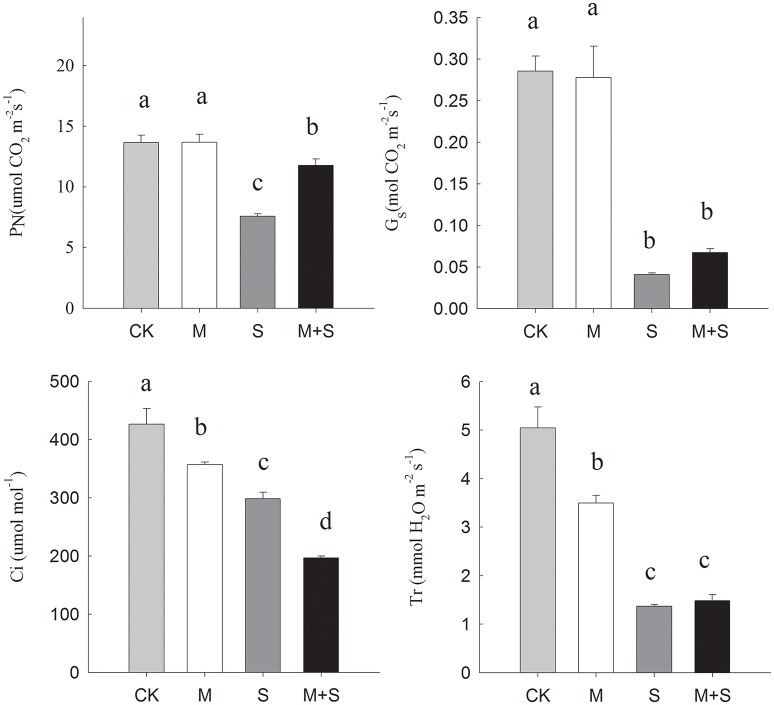
**Effects of NaCl with/without melatonin on gas exchange parameters in tomato seedlings**. PN, net photosynthetic rate; Gs, stomatal conductance; Ci, intercellular CO_2_ concentration; Tr, transpiration. The results were calculated from data for 12-day, salt-stressed leaves. CK, control; M, 150 μM melatonin; S, 150 mM NaCl; M+S, 150 μM melatonin with 150 mM NaCl. Values represent the means ± SE (*n* = 3). Letters indicate significant differences at *P* < 0.05 according to Duncan's multiple range tests.

Salt stress significantly decreased PN, Gs, Ci and Tr compared with the control (Figure 2). However, with exogenous melatonin, the salt stress reductions in PN were mitigated, but melatonin did not significantly mitigate the decreases in Gs and Tr, meanwhile the decrease in Ci was deepened. In the absence of salt stress, melatonin reduced Ci and Tr but did not significantly affect PN or Gs. In summary, over the full period of salt stress treatment, applications of exogenous melatonin increased PN but lowered Ci compared with under salt stress alone.

After 12 days of NaCl treatment the fresh and dry weights of shoot and root were significantly reduced compared with the control (Table 2). However, the addition of exogenous melatonin with the NaCl treatment mitigated the decreases in weight due to NaCl. In the absence of NaCl, melatonin significantly increased root weight compared to the control.

**Table 2:** **Effects of exogenous melatonin on fresh and dry weights in tomato seedlings**.

**Treatments**	**Fresh shoot (g/plant)**	**Fresh root (g/plant)**	**Dry shoot (g/plant)**	**Dry root (g/plant)**
CK	9.1046 ± 0.5106a	2.9924 ± 0.1094b	1.1150 ± 0.0499a	0.2526 ± 0.0134b
M	8.9056 ± 0.2333a	3.4170 ± 0.0690a	1.0964 ± 0.0275a	0.2840 ± 0.0078a
S	3.8452 ± 0.1805c	1.6182 ± 0.1139d	0.5068 ± 0.0129c	0.1564 ± 0.0088d
M+S	4.9316 ± 0.2028b	2.1382 ± 0.0792c	0.6656 ± 0.0324b	0.2068 ± 0.0042c

*Values represent the means ± SE (n = 5). Letters indicate significant differences at P < 0.05 according to Duncan's multiple range tests. Treatments: CK, control; M, 150 μM melatonin; S, 150 mM NaCl; M+S, 150 μM melatonin with 150 mM NaCl*.

### Quantitative reverse transcription (qRT–PCR) assays to analyze expression of some of the key genes in redox regulation

**Figure 3:**
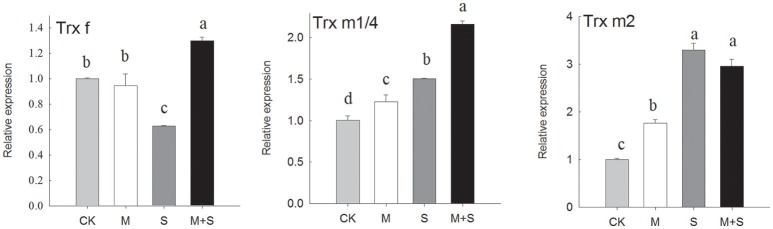
**qRT-PCR analyses of expressions of genes that regulate redox**. Results were analyzed from data for 12-day, salt-stressed leaves. CK, control; M, 150 μM melatonin; S, 150 mM NaCl; M+S, 150 μM melatonin with 150 mM NaCl. qRT-PCR analyses of expressions of TRX f (thioredoxin f), TRX m1/4 (thioredoxin m1/4) and TRX m (thioredoxin m2). Letters indicate significant differences at *P* < 0.05 according to Duncan's multiple range tests.

The expression of some of the key genes of redox regulation in leaves was determined after 12 days of NaCl stress (Figure 3 and Supplementary Figure 1). Moieties examined include: TRXf, TRXm1/4, TRXm2, FTR, NTRC, PRXQ, PRX2E-1, PRX2E-2, 2CPA, and 2CPB.

As shown in Figure 3, the TRXf gene was down-regulated after 12 days of NaCl treatment. When NaCl treated plants also received melatonin, the TRXf gene was up-regulated to a level significantly higher than the control. Melatonin alone did not change the expression of TRXf. However, under NaCl stress, TRXm1/4 showed an approximately 1.5-fold increase in transcription and TRXm2 an approximately 3-fold increase. The salt stressed seedlings also receiving melatonin showed increased gene expression of TRXm1/4 but a similar level of expression of TRXm2. The melatonin treatment in the absence of salt showed a slight increase in gene expression of both TRXm1/4 and TRXm2, compared with the control.

The PRX-related genes were tested (Supplementary Figure 1). NaCl decreased the transcriptional levels of PRXQ-R, PRX2E-1, PRX2E-2, 2CPA but not of 2CPB, with no differences among treatments. Under salt stress and in the presence of melatonin, there was a slight increase in the 2CPA transcript but no significant changes in the other PRX-related genes. Similarly, the expressions of FTR and NTRC were decreased by salt and melatonin had not affect.

In summary, salt stress down-regulated PRXs, FTR, and NTRC both with and without melatonin. While there were different reactions among the TRXs, salt stress down-regulated the expression of TRXf and up-regulated the expressions of TRXm1 and TRXm2. Meanwhile, exogenous melatonin alleviated the transcription level in TRXf and induced TRXm1 under salt stress.

### Effects of exogenous melatonin on ROS levels and D1 protein abundance

**Figure 4:**
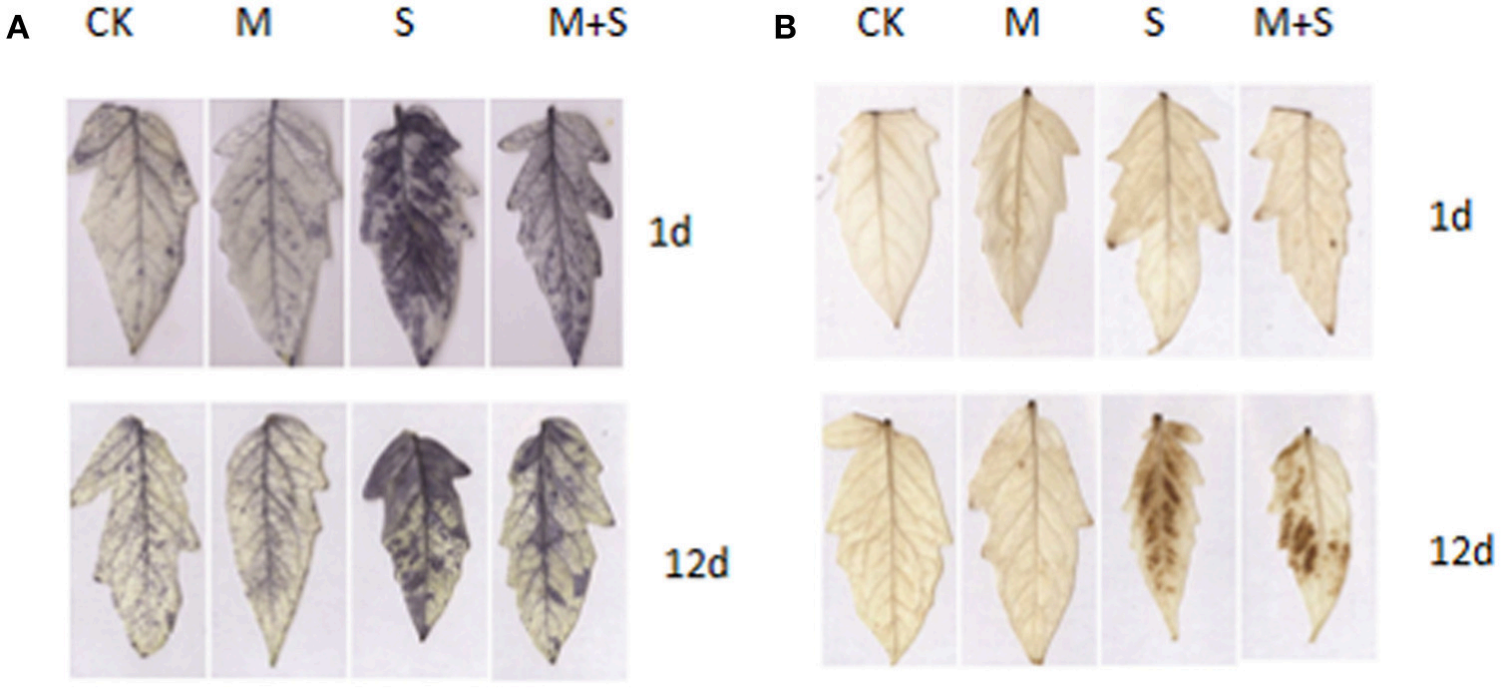
**Exogenous melatonin effects on levels of reactive oxygen species (ROS)**. CK, control; S, 150 mM NaCl; M+S, 150 μM melatonin with 150 mM NaCl. **(A)** Histochemical localization of ${\text{O}}_{2}^{-\cdot }$ by NBT staining after 1 day (top) and 12 days (bottom) of salt treatment; **(B)** Histochemical localization of H_2_O_2_ by DAB staining after 1 day (top) and 12 days (bottom) of salt treatment. CK, control; M, 150 μM melatonin; S, 150 mM NaCl; M+S, 150 μM melatonin with 150 mM NaCl.

Under histochemical staining, ${\text{O}}_{2}^{\cdot -}$ is indicated by dark blue spots (Figure 4A) and H_2_O_2_ is indicated by brown spots (Figure 4B). Significant increases in accumulation of ${\text{O}}_{2}^{\cdot -}$ were noticed in the NaCl stressed leaves, and the number of spots increased with time. Melatonin additions mitigated the salt stress effects reducing the number of spots of ${\text{O}}_{2}^{\cdot -}$ between 1 and 12 days. Significant increases in H_2_O_2_ accumulation were found in the NaCl stressed leaves after 12 days but not after just 1 day. Seedlings incubated with melatonin had suppressed accumulations of H_2_O_2_ after 12 days.

**Figure 5:**
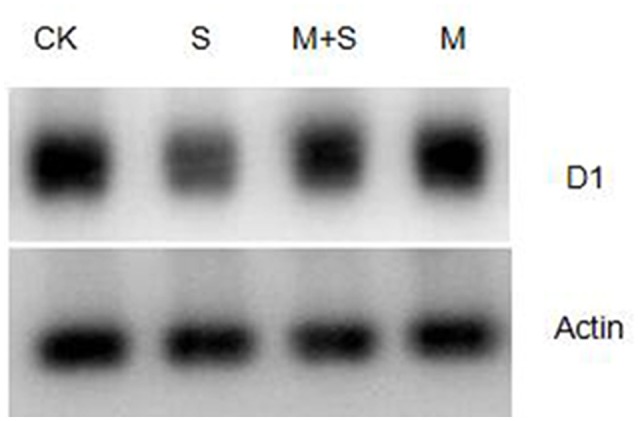
**Immunoblot analyses of D1 protein abundance in tomato leaves**. Leaf extracts from different salt /melatonin treatments after 12 days. Immunoblot analysis of D1 protein using polyclonal antibody (Agrisera, Sweden) and secondary antibody (anti-rabbit IgG horse radish peroxidase conjugated, Agrisera, Sweden). Immunoblotting of actin (ACT, 45 kDa bottom) served as loading controls. CK, control; M, 150 μM melatonin; S, 150 mM NaCl; M+S, 150 μM melatonin with 150 mM NaCl.

The level of D1 protein was analyzed by western blot. Salt stress reduced D1 protein abundance, compared with the control (Figure 5). However, exogenous melatonin suppressed the reduction of D1 protein. Exogenous melatonin alone had no effect on the abundance of D1 protein under control condition.

### Immunoblot analyses of TRXf and TRXm in leaves

**Figure 6:**
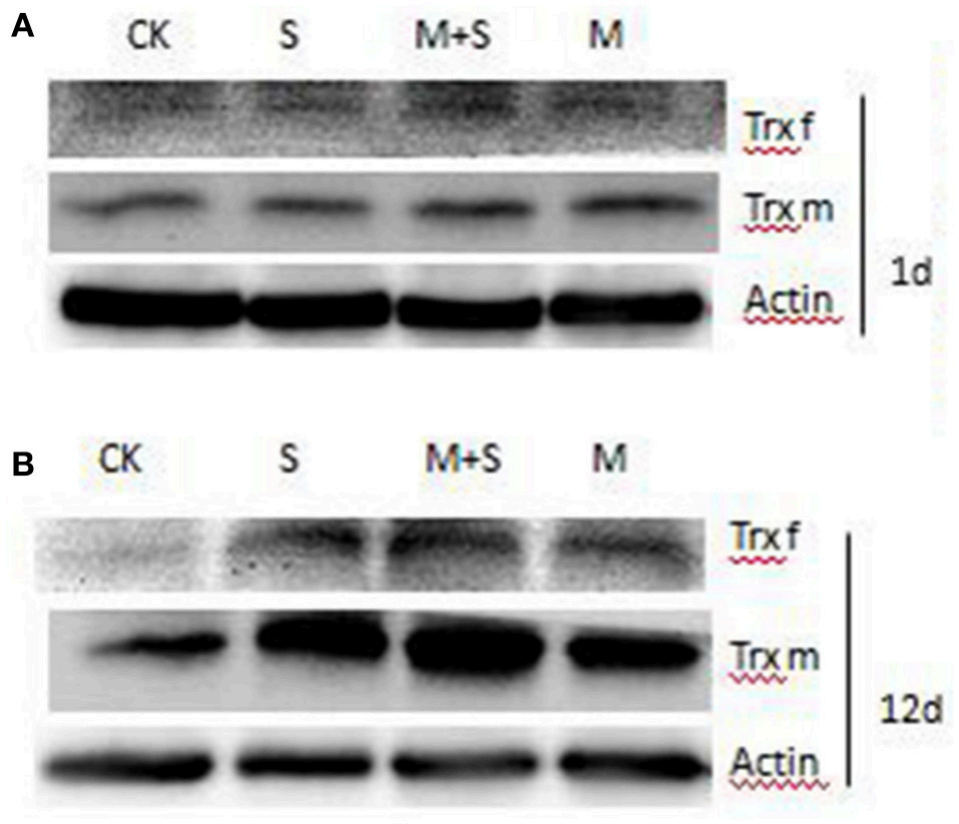
**Immunoblot analyses of TRX f and TRX m in tomato leaves**. Leaf extracts from different treatments after 1 day and after 12 days. Total proteins was analyzed using antibodies raised against pea TRX f and pea TRX m. Immunoblotting of actin (ACT, 45 kDa bottom) served as loading controls. CK, control; M, 150 μM melatonin; S, 150 mM NaCl; M+S, 150 μM melatonin with 150 mM NaCl.

Figure 6 shows that after 1 days of NaCl treatment with/without melatonin, TRX f content did not vary with respect to the control. After 12 days, the TRX f was induced by salt stress and in the presence of melatonin TRXf level was further enhanced. Similarly, TRXm was significantly induced by salt treatment after 12 days. Melatonin also increased the level of TRXm in salt stressed and un-stressed leaves.

## Discussion

Previous studies have shown a beneficial role of melatonin in the regulation of plant growth under NaCl stress (Li et al., 2012; Wang et al., 2016). Here, we confirm this benefit of melatonin on plant growth in tomato seedlings under salt stress (Table 2), and further in regard to the role of melatonin in maintaining photosynthesis (Table 1, Figures 1–6). Compared with the controls, leaves of tomato seedlings under salt stress showed significant reductions in ΦPSII, qP, qL, and Fv′/Fm′ but higher NPQ. All these indicate the inhibition of electron transfer in PSII by salt stress. Still under the salt stress, but in the presence of melatonin, ΦPSII was significantly higher, indicating more energy was used in the photochemistry. However, among the different concentrations of melatonin examined here (1, 50, 100, 150, and 200 μM), alleviation of the effects of salt stress on ΦPSII, qP and qL occurred over the melatonin concentration range from 50 to 150 μM, with 150 μM melatonin being optimal (Table 1). The highest melatonin concentration of 200 μM did not alleviate salt stress, indicating melatonin affects the system differently at different concentrations. The individual behaviors of the various chlorophyll fluorescence parameters, enable the reason for change in ΦPSII to be assessed in relation to electron transport by determining Fv′/Fm′ and qP (Baker, 2008). The ratio Fv′/Fm,′ the efficiency of excitation energy capture by the open PSII reaction center, has also been used to evaluate the contribution of non-photochemical quenching changes in the PSII (Baker, 2008). Generally, increases in NPQ are indicated by decreases in Fv′/Fm.′ In this study, however, there were no changes in Fv′/Fm′ or NPQ with/without melatonin. This suggests no significant effect on changes in non-photochemical quenching by melatonin under salt stress. However, the melatonin treatment also showed higher values for qP and qL, suggesting a greater fraction of maximum PSII efficiency and a greater fraction of open reaction centers, respectively (Kramer et al., 2004). Studies of plants under water stress induced by polyethylene glycol (PEG) treatment, with/without melatonin, yielded similar results—vis. a small change in Fv′/Fm′ and a higher qP. Thus, ΦPSII was higher in the presence of melatonin in the PEG-treated samples than without melatonin (Zhang et al., 2013). Based on these results, we infer that after 12 days of salt stress, exogenous melatonin increased the quantum yield of PSII photochemistry due to a larger number of open reaction centers and an excited PSII reaction center.

Salt stress can affect photosynthesis both via stomatal opening and also via the photosynthetic machinery. Under salt stress, net photosynthetic rate is inhibited by a stomatal limitation factor and/or by non-stomatal limitation factor. The stomatal limitation factor involves reduced stomatal opening, which impedes the diffusion of CO_2_ from the air to the mesophyll (chlorophyll). As a result, the partial pressure of CO_2_ in the leaf is reduced and the photosynthetic rate is slowed. Meanwhile, the non-stomatal limitation factor slows photosynthesis by inhibiting CO_2_ assimilation, including reduced mesophyll conductance, reduced activities of PSII including of the key enzymes of CO_2_ fixation. Thus, the CO_2_ cannot be fully utilized due to a lowered photosynthetic efficiency and so Ci increases. Under salt stress, net photosynthetic rate is inhibited by the stomatal limitation factor and/or by the non-stomatal limitation factor. There were also reductions in PN, Gs and Ci in the salt stressed seedlings. These were caused mainly by the stomatal limitation factor. However, under the salt stress but in the presence of melatonin, PN was increased, there was no change in Gs, and Ci was lower. This may have been caused by a higher CO_2_ assimilation rate at the reduced CO_2_ concentration, indicating regulation of the non-stomatal limitation by melatonin. Previous studies have shown that reduction in photochemical activity is one of the non-stomatal factors that limits photosynthesis (He et al., 2009). Our results on chlorophyll fluorescence (Table 1), partitioning of Absorbed Light Energy on photochemical activity (Figure 1) enhanced by melatonin confirmed the involvement of melatonin in non-stomatal regulation.

In this experiment, the expression of TRXf in tomato leaves was down-regulated under a longer period (12 days) of salt stress, but this effect was reversed by melatonin. Core chloroplastic TRXs, including TRXf, also display decreased expressions in response to salt stress in *Arabidopsis thaliana* (Belin et al., 2015). Salt stress down-regulated the expression of TRXf under longer (5 days) periods of salt stress in the leaves and roots of pea, although there was up-regulation of TRXf after a shorter period (1 day) of salt stress in pea leaves (Fernández-Trijueque et al., 2012). However, taking into account the protein level in our experiment, TRXf was induced by both salt and by melatonin. The TRXf protein level was highest in the salt-stressed samples with melatonin. Post-translational regulation has been suggested for TRXf, due to a lack of correlation between the mRNA and protein levels (Montrichard, 2003; Fernández-Trijueque et al., 2012). The abundance of TRXm protein showed the same behavior being induced both by salt stress and by melatonin (Figure 5B). In this experiment, we also found up-regulated transcripts of TRXm1/4 and TRXm2 in salt-stressed leaves after 12 days. Similarly, TRXm was also induced by oxidative signaling in salt-stressed pea leaves (Fernández-Trijueque et al., 2012), which accumulated higher levels of superoxide anion. This suggests ROS signaling was involved in the expression of TRXm. Although exogenous melatonin resulted in increased levels of TRXm protein under salt stress, we cannot anticipate an effect of melatonin on TRXm1/4 and TRXm2, because of their similarity. This may be a cross action by western blotting and we need further research in this respect.

As is known from previous studies, the AsA-GSH cycle induced by melatonin, together with direct scavenging machanism of ROS by melatonin, enhance the plant tolerance to salt stress (Li et al., 2012) and the other abiotic stresses (Wang et al., 2012; Arnao and Hernandez-Ruiz, 2014; Manchester et al., 2015). In the present study, more efficient ROS scavenging in the presence of melatonin is shown in Figure 4. However, PRX pathways provide an alternative pathway to the water-water cycle in the light, particularly if the AsA-GSH cycle is impaired (Foyer and Shigeoka, 2011). Among the TRXs, it has been suggested that TRXx and TRXm reduce the PRXs (Lemaire et al., 2007). In our study, we examined the expressions of all four PRXs types: 2CPA, 2CPB, PRXQ, and PRX2e. Under salt stress, we observed significant decreases in these. Applications of exogenous melatonin did not induce the expression of three of these PRXs, but did induce 2CPA. Using 12 different *Arabidopsis* microarrays, Dietz et al. (2006) found the expressions of all four types of PRXs decreased under saline conditions—i.e., 2CPA, 2CPB, PRXQ, and PRX2e. Conversely, in the halophyte *E. halophilum*, PRXs expression was up-regulated (Stepien and Johnson, 2009). In the same study, they also compared the halophyte, *E. halophilum* and the glycophyte *Arabidopsis* under saline conditions. They found the rate of linear electron transport (positively correlated with the generation of NADPH and of ATP) was unaffected by salinity in *E. halophilum* but decreased in *Arabidopsis*. This suggests down-regulation of the TRX–PRX system due to insufficient reducing power from linear electron transport. In our study, we also found the up-regulated expression of TRXf and the abundance of TRXf and TRXm gene products melatonin. It was indicated that melatonin may provide an alternative pathway to the water-water cycle in the light, with more potential ability of redox regulation by TRXs.

The activity of PSII depends on the balance between the rate of photodamage to PSII and the rate of damage repair. Thus, photoinhibition becomes apparent only when the rate of photodamage exceeds the rate of repair (Nishiyama et al., 2006). The repair of PSII requires the rapid synthesis of D1 protein. It has been shown that salt inhibits synthesis of the D1 protein due to suppression of the elongation step of translation (Allakhverdiev et al., 2002). This is the first evidence of an effect of melatonin on the translational step of D1 protein under salt stress conditions. Melatonin counteracts the reduction of the *de novo* synthesis of D1 protein by salt stress. Because the elongation factor is sensitive to ROS (Nishiyama and Murata, 2014), the translation machinery may be protected by melatonin. The reduction of ROS levels by melatonin may result both from direct ROS scavenging by melatonin and also via the melatonin-induced antioxidant system. On the other hand, redox regulation is essential for the biogenesis of the photosynthetic apparatus and the maintenance of photosynthetic efficiency (Kieselbach, 2013). The assembly of the PSII complex requires the formation of disulfide bonds in the oxygen-evolving complex (Karamoko et al., 2011). The assembly of D1 proteins into the PSII RC and PSII core complex, depends on the appropriate redox conditions. The induction of TRXf under salt stress by exogenous melatonin, could be more effective in regulating the repair of PSII, which shows more D1 protein and higher photosynthetic activities compared to under salt stress alone.

Based on the mentioned results and analysis, we hypothesize that salt stress severely decreases photosynthesis, at least in part, by inhibiting the repair of the photosynthetic machinery. The molecular mechanisms through which melatonin supports the protective mechanisms of the photosynthetic machinery under salt stress are based on its potential role in regulating the PET chain and redox regulation, including ROS control, and biosynthesis of thiol compounds such as TRXf and TRXm. Mitigation of the salt-induced damage to photosynthesis mediated by melatonin will have positive-going effects on plant growth under salt stress.

## Author contributions

XZ and ZZ conceived and designed the experiment. XZ and HZ carried out the experiment. LH and TD offered technological support, materials and analytical tools. XZ and HZ analyzed the data. XZ wrote the manuscript. KC and FB supervised the experiments and helped to revise the manuscript. All authors have read and have approved the manuscript.

### Conflict of interest statement

The authors declare that the research was conducted in the absence of any commercial or financial relationships that could be construed as a potential conflict of interest.

## Supplementary material

Supplementary Table 1 **Primers for real-time quantitative PCR**.

Supplementary Figure 1 **qRT-PCR analyses of expressions of NTRC (NADPH thioredoxin reductase C), FTR (ferredoxin thioredoxin reductase), PRXQ (peroxiredoxin Q), PRX2E-1 (peroxiredoxin IIE-1), PRX2E-2 (peroxiredoxin IIE-2), 2CPA (2-cys peroxiredoxin A), and 2CPB (2-cys peroxiredoxin)**. Values represent the means ± SE (*n* = 3). Letters indicate significant differences at *P* < 0.05 according to Duncan's multiple range tests. CK, control; S, 150 mM NaCl; M+S, 150 μM melatonin with 150 mM NaCl.

Supplementary Figure 2 **Phylogenetic tree of chloroplastic thioredoxins from ***Solanum lycopersicum, Pisum sativum*** and those identified thioredoxins from ***Arabidopsis*****. The phylogenetic tree was constructed using MEGA 5 with the Neighbor–Joining method. Bootstrap values calculated from 1000 trials are shown at each node. The extent of divergence according to the scale (relative units) is indicated at the bottom.
