# Supplementary material for: Beneficial Roles of Melatonin on Redox Regulation of Photosynthetic Electron Transport and Synthesis of D1 Protein in Tomato Seedlings under Salt Stress
Source: Front Plant Sci. 2016 Nov 30;7:1823. doi: 10.3389/fpls.2016.01823 (PMC5127804; doi:10.3389/fpls.2016.01823)
Supplement: Supplementary Table 1 — Primers for real-time quantitative PCR. [file Table1.PDF]

Table 1. Gene-specific primers designed for quantitative real-time RT-PCR

**Gene**	**Accession numbers**	**Sequence (5’-3’)**
TRX-f	Sl05g056300	F: CACAGTGGTGTGGTCCTTGT; R: TTTGCTAGTGGCCTGTTGTC
TRX-m1/4	Sl12g013810	F: CCGTCGTTGTCTCCTGTTT; R: GCCTCACAGACGATTCTTCC
TRX-m2	Sl10g006970	F: GTCGATCCTCGGCAGTTAAT; R: TTTCCTGAGCTTCACAGACG
NTRC	Sl10g080080	F: GGAGTTGATAACGAGCAAACC; R: ACCCGCATCCACTTCCAT
FTR	Sl02g087230	F: GCAGAAAACGAGACAACGAAG; R: GCAGCGATGAGTAGAAGAAGG
PRX-Q	Sl07g042440	F: AAAGCAGGGGCTCAAGTTG; R: GGATTCCCCACTCTTTCCTAAC
PRX2E-1	Sl10g083650	F: AAGAATCTCCGCCGTCATC; R: CGACTTCTCCACGAAACCAG
PRX2E-2	Sl07g020860	F: TACTGCCTCCCAAGTGTTCTG; R: CCAGTTTCTTTGCCTCCAAC
2CPA	Sl01g007740	F: GATCCTTCGTCGTTCGTGC; R: TCGCTGAAAGCAGTAATCTCTG
2CPB	Sl10g082030	F: TTGTTTCTCGTGTAGCTCGTTC; R: CTTCAGCCTCAAAGTCTGGTG
