# Supplementary figures and images for: Beneficial Roles of Melatonin on Redox Regulation of Photosynthetic Electron Transport and Synthesis of D1 Protein in Tomato Seedlings under Salt Stress
Source: Front Plant Sci. 2016 Nov 30;7:1823. doi: 10.3389/fpls.2016.01823 (PMC5127804; doi:10.3389/fpls.2016.01823)

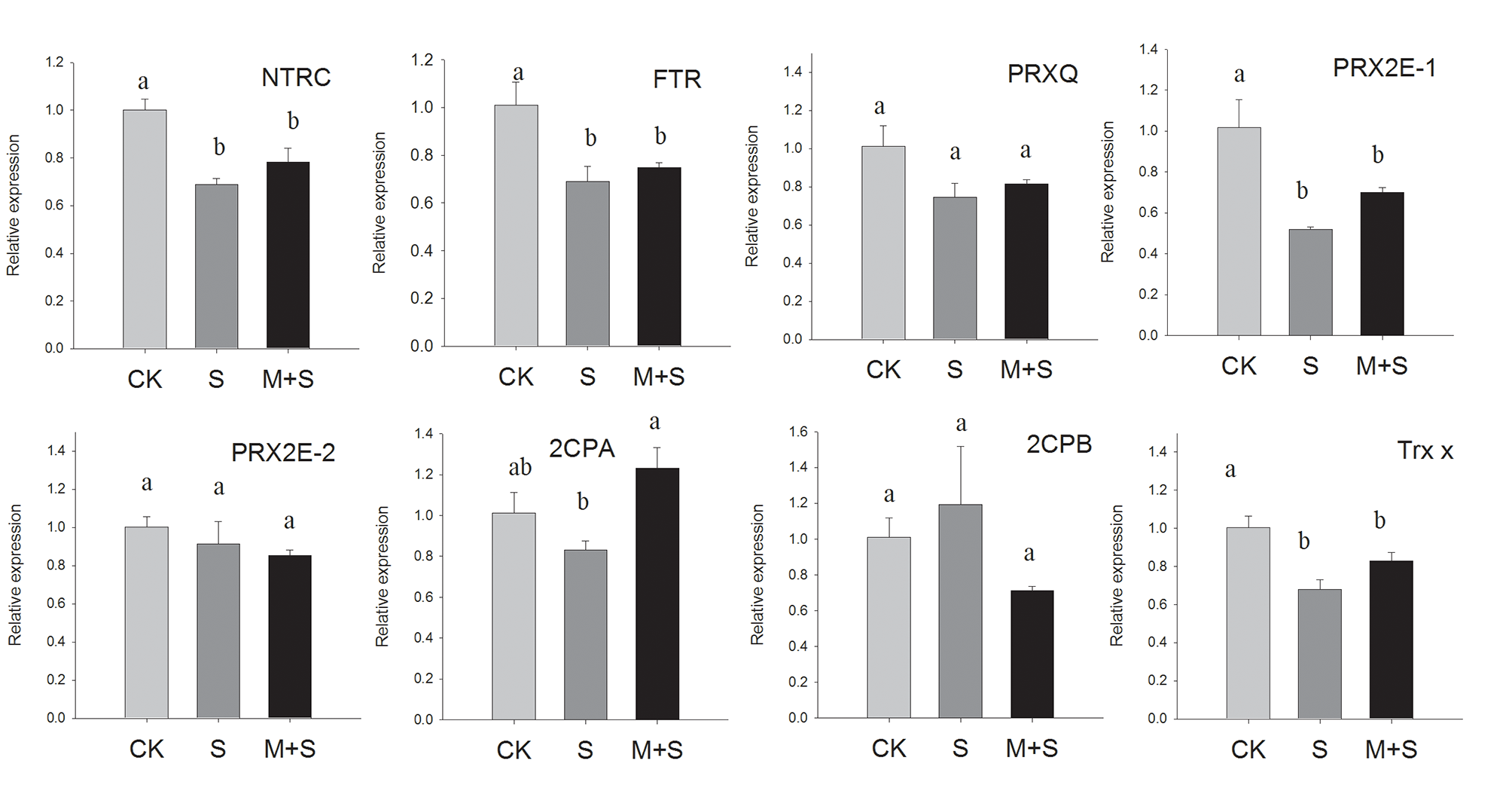

Supplement: Supplementary Figure 1 — qRT-PCR analyses of expressions of NTRC (NADPH thioredoxin reductase C), FTR (ferredoxin thioredoxin reductase), PRXQ (peroxiredoxin Q), PRX2E-1 (peroxiredoxin IIE-1), PRX2E-2 (peroxiredoxin IIE-2), 2CPA (2-cys peroxiredoxin A), and 2CPB (2-cys peroxiredoxin). Values represent the means ± SE (n = 3). Letters indicate significant differences at P < 0.05 according to Duncan's multiple range tests. CK, control; S, 150 mM NaCl; M+S, 150 μM melatonin with 150 mM NaCl. [file Image1.TIF]

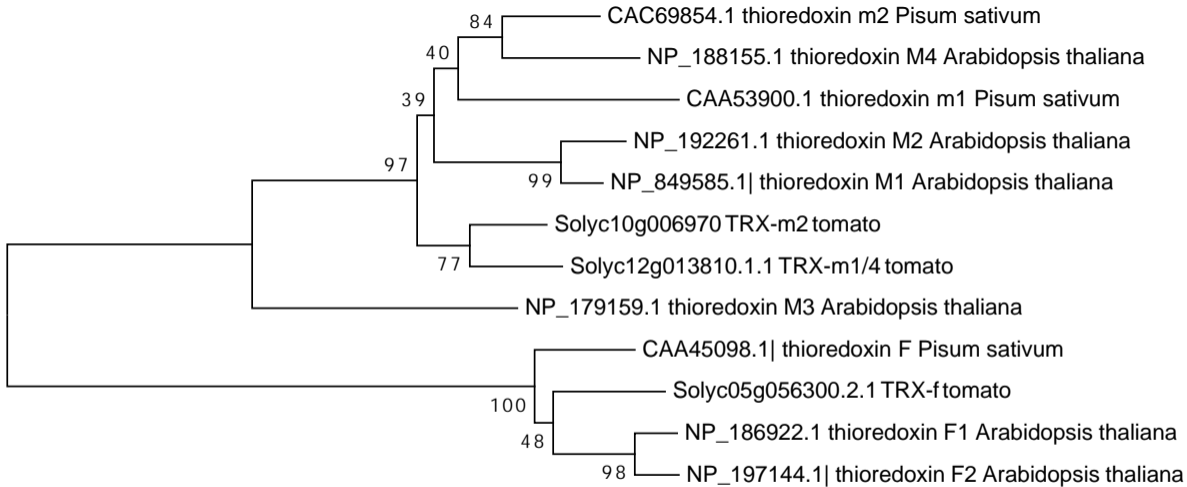

0.2

Supplement: Supplementary Figure 2 — Phylogenetic tree of chloroplastic thioredoxins from Solanum lycopersicum, Pisum sativum and those identified thioredoxins from Arabidopsis. The phylogenetic tree was constructed using MEGA 5 with the Neighbor–Joining method. Bootstrap values calculated from 1000 trials are shown at each node. The extent of divergence according to the scale (relative units) is indicated at the bottom. [file Image2.PDF]
